# Statement of Retraction: Beyond creatinine: diagnostic accuracy of emerging biomarkers for AKI in the ICU – a systematic review

**DOI:** 10.1080/0886022X.2026.2727020

**Published:** 2026-09-02

We, the Editors and Publisher of *Renal Failure*, have retracted the following article:

Matarneh, A., Akkari, A., Sardar, S., Salameh, O., Dauleh, M., Matarneh, B., … Ghahramani, N. (2025). Beyond creatinine: diagnostic accuracy of emerging biomarkers for AKI in the ICU – a systematic review. *Renal Failure*, *47*(1). https://doi.org/10.1080/0886022X.2025.2556295

Since publication, concerns were identified regarding the relevance and validity of the references used in this article. When approached for an explanation, the authors agreed with the concerns and requested a retraction. As the Editor and Publisher agreed with these identified concerns, in accordance with the journal editorial policies, all parties have agreed to retract the article to ensure correction of the scholarly record.

As verifying the validity of published work is core to the integrity of the scholarly record, we are therefore retracting the article. The corresponding author has been informed.

We have been informed in our decision-making by our editorial policies and the COPE guidelines.

The retracted article will remain online to maintain the scholarly record, but it will be digitally watermarked on each page as ‘Retracted’.

